# Supplementary material for: Protein interaction networks define the genetic architecture of preterm birth
Source: Sci Rep. 2022 Jan 10;12:438. doi: 10.1038/s41598-021-03427-0 (PMC8748950; doi:10.1038/s41598-021-03427-0)
Supplement: Supplementary file 1 — Supplementary Tables. [file 41598_2021_3427_MOESM1_ESM.docx]

**Supplementary Information**

**Protein Interaction Networks Define the Genetic Architecture of Preterm Birth**

Alper Uzun,^ Jessica S. Schuster,^ Joan Stabila, Valeria Zarate, George A. Tollefson,

Anthony Agudelo, Prachi Kothiyal, Wendy S.W. Wong, James Padbury^

**Supplemental Table 1.** Functional annotation and variant numbers corresponding to pathogenicity and genotype filtering.

| **Function**  **“ref Gene”** | ***DP>10** | **With DP>10 &**  **Genotype Testing** | **With DP>10 &**  **Pathogenicity Testing** |
| --- | --- | --- | --- |
| upstream | 218 | 5 | 0 |
| UTR5 | 737 | 27 | 0 |
| intronic | 30266 | 855 | 0 |
| exonic | 3608 | 126 | 264 |
| UTR3 | 3822 | 102 | 0 |
| downstream | 235 | 3 | 0 |
| ncRNA_exonic | 105 | 8 | 0 |
| splicing | 11 | 0 | 0 |
| ncRNA_intronic | 457 | 5 | 0 |
| exonic; splicing | 2 | 0 | 0 |
| upstream; downstream | 11 | 0 | 0 |
| **TOTAL** | **39472** | **1131** | **264** |

**Supplemental Table 2**. Genes and group membership of the genes from the layered network graph of the two case dominated preterm birth clusters (A and B) and the four case dominated clusters from the replication cohort (A’, B’, C, D’)
